# Varieties of visual navigation in insects

**DOI:** 10.1007/s10071-022-01720-7

**Published:** 2022-11-28

**Authors:** Cody A. Freas, Marcia L. Spetch

**Affiliations:** 1 Department of Psychology, University of Alberta, Edmonton, AB Canada; 2 School of Natural Sciences, Macquarie University, Sydney, NSW Australia

**Keywords:** Celestial compass, Orientation, Migration, Wind compensation, Landmarks, Panorama

## Abstract

The behaviours and cognitive mechanisms animals use to orient, navigate, and remember spatial locations exemplify how cognitive abilities have evolved to suit a number of different mobile lifestyles and habitats. While spatial cognition observed in vertebrates has been well characterised in recent decades, of no less interest are the great strides that have also been made in characterizing and understanding the behavioural and cognitive basis of orientation and navigation in invertebrate models and in particular insects. Insects are known to exhibit remarkable spatial cognitive abilities and are able to successfully migrate over long distances or pinpoint known locations relying on multiple navigational strategies similar to those found in vertebrate models—all while operating under the constraint of relatively limited neural architectures. Insect orientation and navigation systems are often tailored to each species’ ecology, yet common mechanistic principles can be observed repeatedly. Of these, reliance on visual cues is observed across a wide number of insect groups. In this review, we characterise some of the behavioural strategies used by insects to solve navigational problems, including orientation over short-distances, migratory heading maintenance over long distances, and homing behaviours to known locations. We describe behavioural research using examples from a few well-studied insect species to illustrate how visual cues are used in navigation and how they interact with non-visual cues and strategies.

## Introduction

In the inaugural issue of *Animal Cognition*, Czeschlik (1998) wrote the following:

“The aim of this new journal is to establish the course of the evolution of “intelligence”, of the mechanisms, functions, and adaptive value of basic and complex cognitive abilities — the evolution of intelligent behaviour and intelligent systems from invertebrates to humans”.

In the 25 years since this opening issue was published, the journal (and the field of comparative cognition in general), has made great progress towards this aspiration, with considerable advances in understanding the mechanisms and processes underlying many types of intelligent behaviours in a wide range of species. One broadly important example of evolved intelligence is the ability to orient, navigate and remember places in the world. Over the past few decades, extensive progress has been made in characterising spatial memory and navigation in vertebrates (for reviews see Cheng and Spetch 1998; Cheng et al. 2013; Kelly and Spetch 2012; Tommassi et al. 2012). The ability to orient oneself and maintain a desired heading to reach goal locations is a fundamental and recurring challenge that mobile animals face. Yet invertebrate animals also exhibit spectacular spatial cognitive abilities and can accomplish navigational feats quite similar to those of vertebrate navigators using analogous navigational strategies (Chapman et al. 2011; Cheng 2022; Freas and Cheng 2022).

**Fig. 1:**
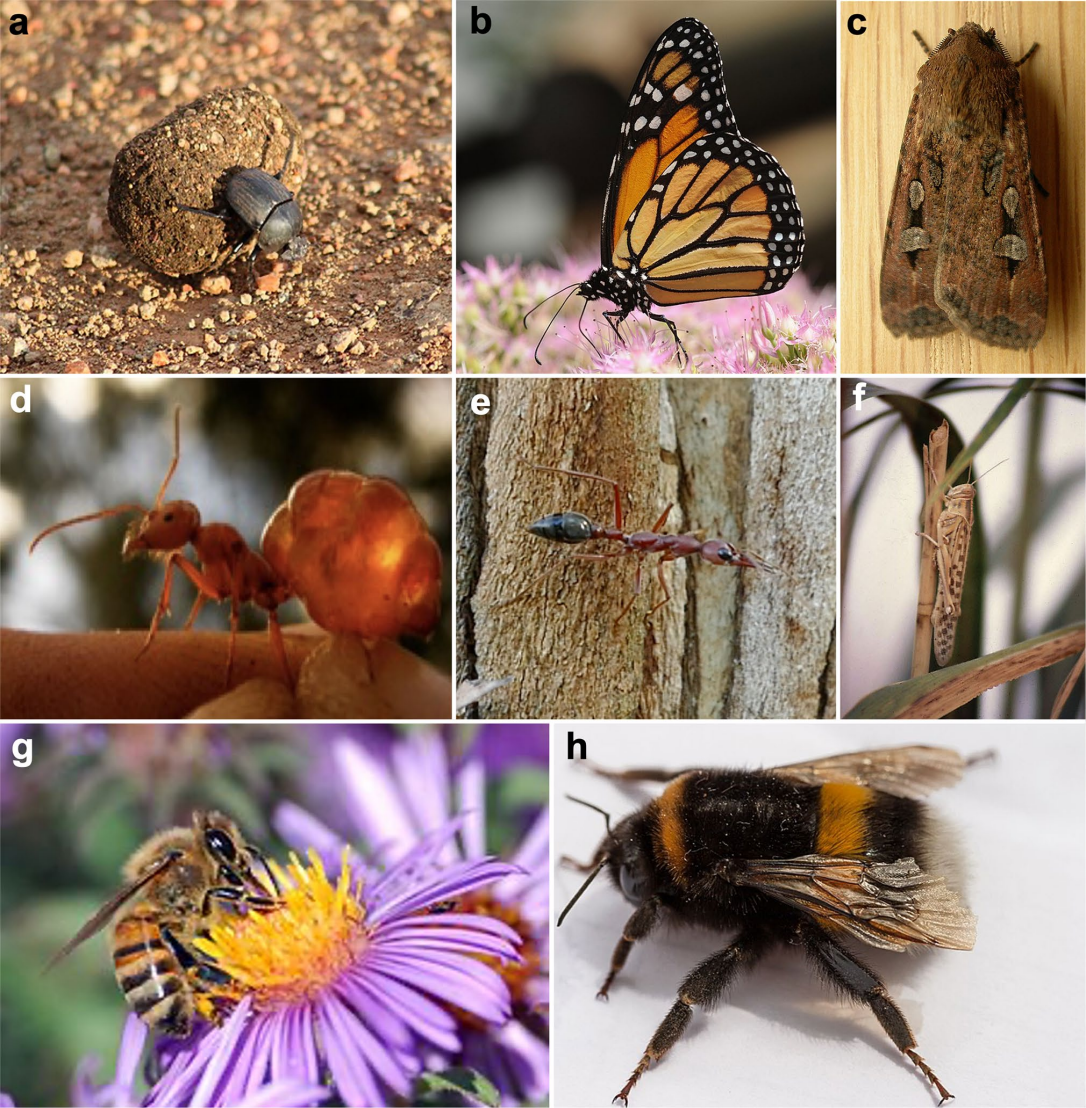
Many insect groups contain members that are well studied for their orientation and navigational abilities. **a** The dung beetle, *Scarabaeus (Kheper) lamarcki.* **b** The monarch butterfly, *Danaus plexippus.* **c** The bogong moth, *Agrotis infusa*. **d** The red honey ant**,** *Melophorus bagoti*. **e** The Australian bull ant, *Myrmecia midas*. **f** The desert locust, *Schistocerca gregaria*. **g** The European honey bee, *Apis mellifera.* **h** The buff-tailed bumblebee, *Bombus terrestris.* Sources: **a** https://commons.wikimedia.org/wiki/File:Dung_beetle_(12593889274).jpg Author: flowcomm. License: https://creativecommons.org/licenses/by-sa/2.0/deed.en. **b** https://commons.wikimedia.org/wiki/File:ComputerHotline_-_Danaus_plexippus_(by)_(3).jpg Author: Thomas Bresson. License: https://creativecommons.org/licenses/by-sa/2.0/deed.en. **c** https://commons.wikimedia.org/wiki/File:Agrotis_infusa.jpg Author: Donald Hobern. License: https://creativecommons.org/licenses/by-sa/2.0/deed.en**d** Author: Patrick Schultheiss (with permission) **e** Author: Cody Freas **f** https://commons.wikimedia.org/wiki/File:Schistocerca_gregaria _solitary.jpg Author: Christiaan Kooyman License: **g** https://commons.wikimedia.org/wiki/File:European_honey_bee_extracts_nectar.jpg Author: John Severns License: https://creativecommons.org/publicdomain/zero/1.0/deed.en**h** https://commons.wikimedia.org/wiki/File:Bombus_terrestris.jpg Author: Marco Almbauer License: https://creativecommons.org/publicdomain/zero/1.0/deed.en

Many insects lead mobile lifestyles, traversing within and between environments during their daily lives (Fig. 1). These small navigators constantly need to accurately set headings and travel between goal locations while foraging, migrating, finding mates, during territorial defence or returning to their nest or home. Yet despite their tiny brains, insects are remarkably adept at both recognising their current position as well as setting desired headings. The breadth of spatial scales across which insects navigate is immense, from migratory journeys spanning thousands of kilometers across continents to short foraging journeys of only a few meters. Insects provide interesting systems for studying spatial behaviour for several reasons. First, they comprise a huge number of species and inhabit a vast range of ecosystems. This makes them ideal for comparative research that aims to understand the evolution and ecological determinants of spatial cognition (Cheng et al. 2014). Second, insects display a wide array of behaviours that are exquisitely adapted to the problems of orienting, navigating, and finding locations. Additionally, while these behaviours are often complex, flexible, and impressive, they are accomplished with a toolkit of simple sensory and behavioural mechanisms (Büehlmann et al. 2020; Cheng et al. 2009; Freas and Cheng 2022; Wehner 2020). Third, because of their small size and large numbers, research with insects lends itself more readily to experimental investigations, especially when characterising long-distance navigation. Fourth, the neural architecture underlying insect behaviour is simpler than that of birds or mammals. This not only makes the goal of understanding the neural underpinnings of navigational behaviour more tractable (e.g., Le Moël and Wystrach 2020; Warren et al. 2019), but also makes research on insect navigation appealing to researchers in artificial intelligence (e.g., de Croon et al. 2022).

Orientation and navigational behaviours in insects rely on each species’ specialised sensory ecology, which involves the weighting and interaction of a number of concurrently running guidance systems (Büehlmann et al. 2020; Wehner 2020). Despite these guidance systems being tailored to each species, common mechanistic principles can be observed across a wide array of insect species (as well as in vertebrates), with these navigational systems described as goal-directed servomechanisms (i.e., self-regulatory control mechanisms operated by negative feedback; Cheng 2022; Freas and Cheng 2022). Goal locations can be represented in a variety of ways, including learned cues acquired during previous trips, innate desired compass directions, or attractive cue gradients to reach migratory destinations. These representations classify the goal as an end state with navigational systems moving animals in ways that reduce the differences or error between the cues at their current location and the goal site (Cheng 2022). Navigational servomechanisms operate by altering the animal’s course to reduce the amount of error between these locations (Cheng 2022). More recently, these navigational servomechanisms are now believed to operate on oscillators, or regularly cycling behaviours (for a detailed review of these oscillators see Cheng 2022).

**Fig. 2:**
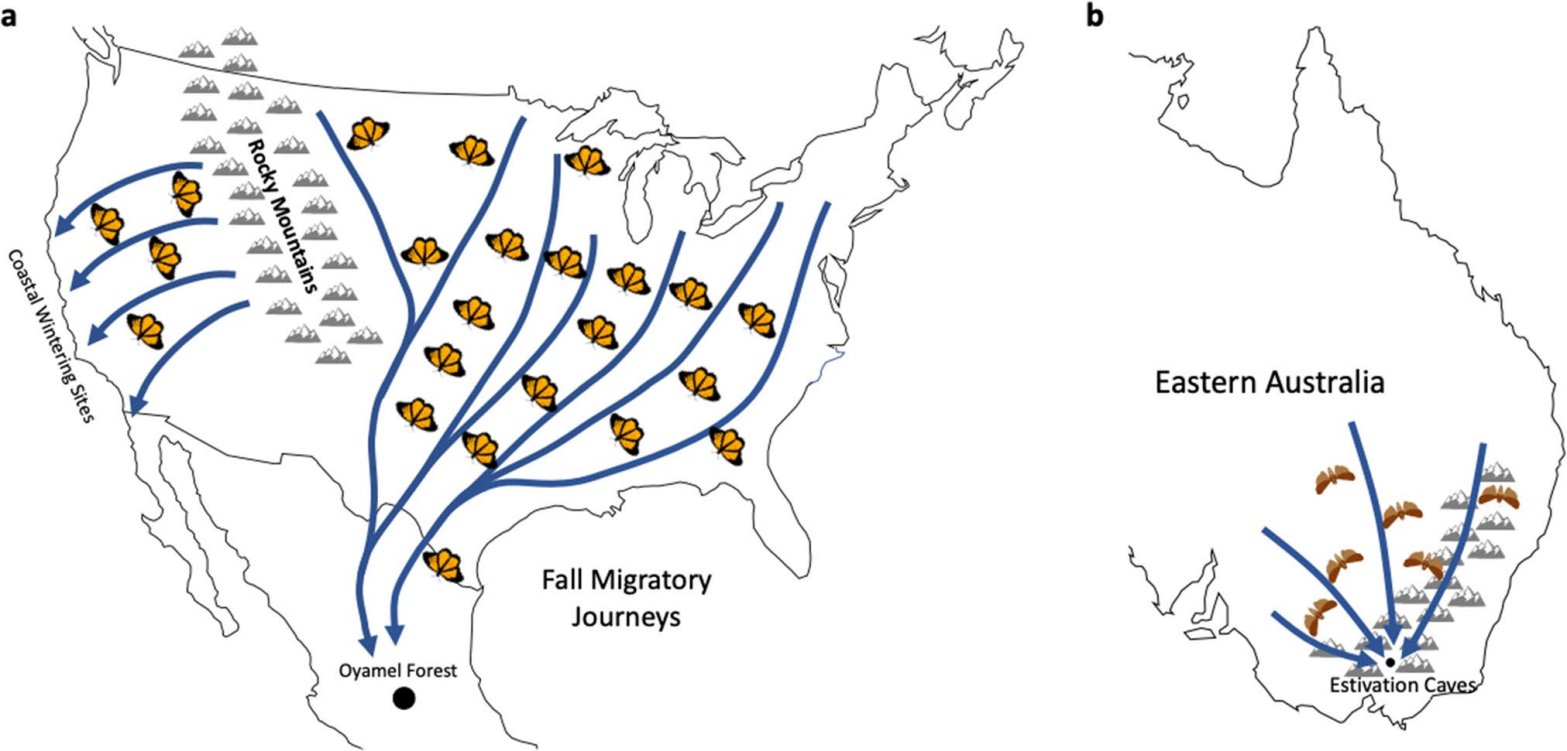
Diagrams of the migration pathways of two lepidopterans to specific hibernation/estivation sites. **a** In North America, the eastern population of Monarch butterflies travel from southern Canada and the northern United States to hibernate in the Mexican Oyamel Fir forest (Brower 1996). Populations west of the Rocky Mountains migrate to the Pacific coast of California where coastal microclimates are suitable for over-wintering hibernation. **b** In Eastern Australia, Bogong moths conduct yearly migratory journeys along the Australian Alps to mountain caves where they estivate over the hot summer months (Warrant et al. 2016)

These guidance systems rely on both external and internal cue sets across several sensory modalities to both denote the goal location as well as determine the individual’s current position, with visual cues being prominently used across many insect species (Büehlmann et al. 2020; Freas and Cheng 2022; Heinze et al. 2018; Schultheiss et al. 2020; Wehner 2020). Here, we review some of the behavioural literature on visually-mediated spatial cognition in insects. Our review is not meant to be exhaustive but instead illustrates some of the navigational problems insects face and the behavioural and cognitive mechanisms that have been identified to solve these problems in a select set of model species. We focus on three general types of navigational problems. First is the problem of maintaining straight-line orientation over short distances, either with or without a specified goal. The second is heading maintenance over long distances, either for dispersal without a specified goal direction, or during migratory journeys (Fig. 2) when the goal location is beyond the navigator's sensory range and their navigational knowledge. The final problem is that of homing, in which a navigator travels to a specific goal site, often a known resource location, and then returns to a starting location, typically their nest. Homing often involves more complex mechanisms with the navigator recalling memories of cues learned during previous trips to these sites.

**Fig. 3:**
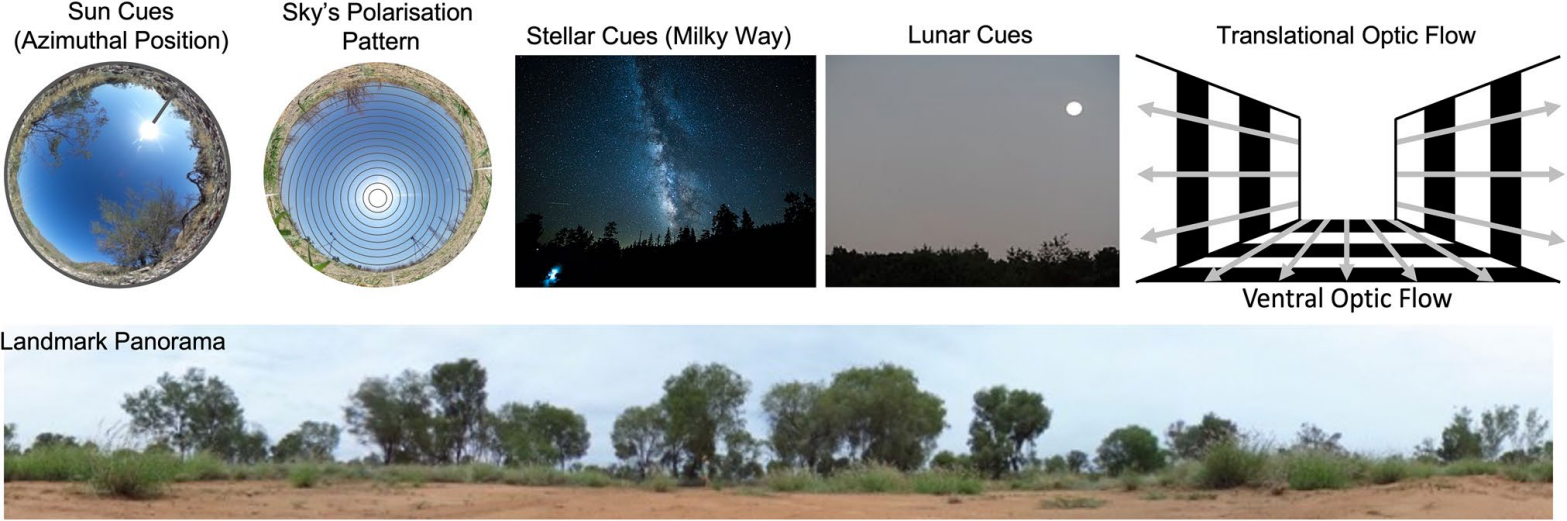
Diagrams of some of the major visual cue sources for orientation and homing in insects. The polarisation pattern in the sky corresponds polarised light in concentric circles around the sun. Sources: Sun cues—Author: Cody Freas; Sky’s Polarisation—Author: Cody Freas; Milkyway—Author: Jakub Gorajek License: https://creativecommons.org/publicdomain/zero/1.0/deed.en; Lunar Cues—Author: Mark Buckawicki License: https://creativecommons.org/publicdomain/zero/1.0/deed.en; Optic Flow – Author: Cody Freas; Landmark Panorama – Author: Cody Freas

For each navigational problem, we present results from a select set of well-studied model species across a range of insect orders to provide an in depth look at the strategies used. For some excellent reviews of navigation in other insects not reviewed here and discussions of neural mechanisms see Warren et al. (2019), Webb and Wystrach (2016) and Heinze (2017). The research we review here illustrates that a broad range of spatial challenges that insects face are solved, at least in part, through the use of the available visual cues (Fig. 3) present across a variety of environments.

## Maintaining orientation over short distances

Many insects show strategies to maintain a consistent orientation over short distances. In some cases, the direction of orientation is random with no specific goal, and in other cases the orientation is in a specific goal direction.

### Goalless orientation

The behaviours and strategies used to orient in a consistent but random direction are best exemplified through the extensive research on dung beetles (see reviews by Dacke et al. 2021; Warrant and Dacke 2016; Fig. 1a), with much of this research focusing on how the diurnal *Scarabaeus (Kheper) lamarcki* and the nocturnal *Scarabaeus satyrus* steer straight when rolling dung away from a dung pat. These beetles roll the dung away to protect it from competitors and then bury it for future use. The behaviour has no specific destination but maintains a consistent direction, maximizing the distance from the collection site.

Despite the apparent simplicity of this orientation behaviour—namely moving in a straight line in a random direction—the mechanisms are fascinating and sophisticated. The consistency of direction is guided primarily by a visually based celestial compass. This compass can rely on a variety of celestial cues and in dung beetles, experimental research has shown the use of the sun (Byrne et al. 2003), the moon (Dacke et al. 2004), and the stars (Dacke et al. 2013), as well as gradients of light and polarisation patterns around the sun or moon (see review by Dacke et al. 2021, Fig. 3). Most impressive is the adaptability and flexibility of the cue use. For example, the sun is typically the dominant cue for orientation, and this has been shown by shifts in orientation when the sun’s position is altered by mirrors, setting it in conflict with other celestial cues (Dacke et al. 2014). However, when the view of the sun is experimentally blocked (e.g., by a board), then the beetles will shift their orientation in response to shifts in the e-vector of polarised light (el Jundi et al. 2014). Moreover, the beetles will shift to using wind direction if sky cues are unreliable, such as when the sun is close to its zenith. For example, experimental studies conducted in a controlled indoor arena have shown that dung beetles can use directional information from the sun and wind and combine the information in a weighted manner (Dacke et al. 2019).

In a recent study, Khaldy et al. (2022) demonstrated Bayesian integration of celestial cues in dung beetle orientation. They tested *Scarabaeus (Kheper) lamarcki* in a controlled indoor arena and showed that the beetles could maintain directional orientation in their dung rolling behaviour with either a green light source that served as an artificial ersatz sun or with an artificial polarised light source. When each cue was at full intensity and presented alone, shifting of the ersatz sun, or rotating the polarisation pattern resulted in corresponding shifts in behaviour. When they were presented together and shifted to produce a conflict, the weight given to each depended on the strength of the cues. The ersatz sun dominated orientation when it was presented at a higher intensity and the percentage of polarisation was low. When the polarisation cue was increased to 100% polarised light (which is higher than the maximum of 80% polarisation found in nature), the polarisation pattern dominated orientation. However, when the polarisation pattern was set to 64%, the beetles showed partial shifts (e.g., 45°) in response to 90° shifts of the sun. Thus, the beetles showed Bayesian averaging of the cues that depended on their reliability, similar to that which has been seen in ants (e.g., Legge et al. 2014; Wystrach et al. 2015) and other species (e.g., Cheng et al. 2007).

Tribes of dung beetles living in different habitats have provided an excellent opportunity for comparative investigations of the match between visual ecology and use of visual cues for orientation. The *Scarabaeini* tribe lives in open habitats and has been found to rely primarily on the sun compass (Fig. 3) whereas the *Sisyphini* tribe lives in habitats that are closed in by trees or tall grass and has been shown to rely primarily on polarised light (Fig. 3). A third tribe, *Gymnopleurini*, also lives in open habitats but weights sun and polarised light equally. In a comparative study, Khaldy et al. (2021) collected beetles from these three tribes and tested their orientation strategies. Individuals within each tribe showed similar cue weighting but the tribes differed from each other and matched the patterns seen in their respective natural habitats. Interestingly, cue weighting was dynamic, and the beetles were able to maintain straight line orientation in the absence of their preferred cue.

In summary, even the seemingly simple task of orientating consistently in a random direction can entail a rather sophisticated set of strategies, including use of celestial cues to maintain direction, the ability to flexibly combine various celestial and non-celestial cues, the ability to weight cues in a Bayesian fashion depending on reliability, and weighting of cues that is both ecological adapted to the home environment but dynamic to allow orientation in the absence of preferred cues.

### Goal-directed orientation

The ability to orient towards a specific direction or goal area over relatively short distances has been studied in many insect species. We will illustrate the use of visual cues for this ability with examples from pest beetles and beach dwelling beetles. Considerable research has been directed at how pest beetles such as weevils orient towards hosts, mates, or shelter locations, often with the goal of identifying ways to control or change their behaviour. Beach dwelling beetles living in coastal habitats have been studied for their ability to orient towards preferred zones in habitats that are constantly changing due to tidal fluctuations, as well as how they recover their preferred zone after displacement.

Many pest beetles use visual cues together with odour plumes to orient towards host plants (de Jonge 2021). For example, the mountain pine beetle, *Dendroctonus ponderosae*, uses both olfactory and visual cues to orient towards host plants, with visual cues augmenting olfactory cues at close range (Campbell and Borden 2006). Switching from olfactory-guided to visual-guided orientation has also been shown in the tropical root weevil, *Diaprepes abbreviates* (Otálora-Luna et al. 2013). In addition to showing that the weevils were attracted to certain wavelengths and intensities Otálora-Luna et al. showed that attraction to the odours of a host was overridden by the presence of green light. Using a choice task, Hausmann et al. (2004) showed that apple blossom weevils, *Anthonomus pomorum*, have a trichromatic visual system and are attracted to certain wavelengths. Moreover, their attraction to the contrast level of a silhouette is modulated by the background wavelength.

Several species of ladybeetles have been shown to use visual cues for orientation to a goal. For example, orientation in the Asian ladybeetle *Harmonia axyridis* (Pallas), an introduced species common in agricultural regions in North America, has been studied both because it is a pest in autumn when large numbers of these beetles infest buildings to overwinter, and because it is a valuable biological control organism that consumes large quantities of aphids. Nalepa et al. (2005) showed that the ladybeetles searching for overwintering sites are attracted to high visual contrast, a common feature on many buildings due to painted trim and shadows from architectural features. Research on ladybeetles’ attraction to aphid-infested plants has shown a role for learning. Specifically, Wang et al. (2015) found that naïve foraging ladybeetles *Propylaea japonica* (Wang et al. 2015) showed no ability to discriminate between aphid-infested and non-infested cotton plants, whereas beetles given experience foraging for aphids were attracted to plants with olfactory cues of aphid infestation and visual cues of aphid infestation enhanced this attraction.

Some beach-dwelling beetles have been found to orient to preferred zones using combinations of visual cues. For example, Colombini et al. (1994) showed that two populations of the gazelle beetle *Eurynebria complanata*, one living in Italy and one in France, used a time-compensated sun compass for orientation, but the compass orientation was strongly influenced by negative phototaxis. Landmark cues provided by the skyline appeared to work in conjunction with the sun compass and reduce errors from the negative phototaxis.

In summary, visual cues are used in many instances of short-distance orientation towards a goal area as illustrated by examples with beetles. The visual cues do not always act as a simple attractant. Instead, information from different types of visual cues such as celestial compass cues and landmarks, or wavelength and silhouettes, interacts, or visual cues are used in combination with information from other modalities such as olfactory cues. There is some evidence that learning can play a role in the development of attraction to cues.

## Long-distance orientation and navigation

Many insect species, particularly flying species, travel long distances, either to disperse from an area or to seasonally migrate to more hospitable habitats. Even when dispersing in an arbitrary direction, maintaining a consistent bearing is critical and visual cues, most commonly celestial in nature, are often used. In migration, the orientation is in a specific direction but cues originating from the destination are beyond the sensory range. Species which conduct migratory journeys are common in all animal groups and insects are no exception, with butterflies, moths, dragonflies, flies, locusts, and bees all containing migratory members (e.g., Aryal 2019; Chapman et al. 2015; Dickinson 2014; Dingle 2014; Dingle and Drake 2007; Fijen 2021; Gao et al. 2020). For example, despite their small size, fruit flies, *Drosophila melanogaster*, are capable of long-distance navigation (Dickinson 2014; Warren et al. 2019). Much like the dung beetle, these fruit flies initially depart in a random direction, but they use both polarised light patterns and the position of the sun to maintain a consistent direction (Warran et al. 2018).

Yet migratory journeys in insects are often directed. Each year, billions of flying insects make long journeys in seasonal migrations, often over multiple generations either to reach sites with more abundant resources, a reproductive habitat, or a specific hibernation refuge. As individual migrants typically only undertake part of the journey, with the remaining stages to be conducted by their offspring, these navigators cannot rely on learned cues from previous trips. Thus, insect migrants rely on innately set compass cues, such as a celestial compass, to stay on course rather than updating estimates of their current position relative to the goal location. This leaves navigators which rely primarily on a compass heading alone, such as the Monarch butterfly (Fig. 1b) following a sun based compass, prone to making displacement errors, as they are unable to correctly update their heading direction after large-scale experimental displacements off their migration path (Mouritsen et al. 2013a; b). Yet under natural displacements (i.e., wind gusts), other navigational systems can often act to correct smaller directional errors (Chapman et al. 2010; Srygley and Dudley 2008). Additionally, migrating insects can use different navigational strategies at distinct portions of their migratory journeys.

Lepidopterans in particular are famous for migrations on a continental scale, with painted ladies (*Vanessa cardui*), Bogong moths (*Agrotis infusa*, Fig. 1c) and Monarch butterflies (*Danaus plexippus*) travelling thousands of miles between their seasonal ranges (Fig. 2). The North American common green darner dragonfly (*Anax junius*) makes similar seasonal journeys, travelling over multiple generations between wintering ranges around the Gulf of Mexico and their summer ranges in the northern U.S. and southern Canada (Hallworth et al. 2018; Wikelski et al. 2006), while the wandering glider dragonfly (*Pantala flavescens*) makes transoceanic journeys across the Indian Ocean, migrating between India and eastern Africa (Anderson 2009; Hedlund et al. 2021).

Fully passive migration in insects appears rare, though many high-altitude migrants do rely heavily on favourable wind directions to reach their destinations. Both high- and low-altitude migrants show clear evidence of active transport with regards to their direction heading, able to dictate their flight direction relative to the wind as well as compensate for cross-wind-related drift through a combination of visual and non-visual based mechanisms (Chapman et al. 2008a, b; Chapman et al. 2015; Reynolds et al. 2010). Given insects are actively navigating during migration, they must rely on the available cues in order to set their headings and reach their goal location. Mouritsen (2018) categorised long distance migration into three stages, during which migrants will alter their navigational strategies and the cues they employ based on cue availability: a long-distance orientation phase, a narrowing phase, and a pinpointing phase. During most of the journey, migrants conduct long-distance orientation, setting a heading direction based on the visual cues present in the celestial compass; yet other cues may also be employed either separately or concurrently, such as visual landmarks and geomagnetic cues (Dreyer et al. 2018b). During long-distance orientation, visual terrestrial cues play less of a role as navigators cannot rely on learned landmarks, especially in high-altitude migrants (yet see wind compensation via optic flow); however, visual cues, such as the celestial compass or prominent visual landmarks such as mountain ranges, can still be critically important for some high-altitude migrating species (discussed more below). Currently, there is little evidence that migrants maintain large-scale distance estimates of their journey via ‘optic-flow’, where distance estimates are calculated via the visual motion passing by the insect’s retina (Fig. 3). This holds particularly true for high-altitude nocturnal migrants, where ground distance and low ambient light levels make terrestrial cue acquisition difficult. Yet as noted above, migrants need to compensate for unfavourable wind directions, lest they be blown off course. Optic-flow-based compensation for drift due to the wind does appear in low-altitude migrating tropical butterflies and dragonflies while high-altitude migrants such as nocturnal moths appear to rely on non-visual mechanisms, responding to wind turbulence, to compensate for crosswind drift (Chapman et al. 2011, 2015).

In the following sections we present several types of visual cue use, using a select set of examples from well-studied migratory insects including the Monarch butterfly, the Bogong moth, and the desert locust*.*

### Celestial compass use to maintain headings

Similar to navigation and migration in mammals and birds, many migrating insects appear to rely on a time-compensated celestial compass that is primarily informed by the sun. Likely the most famous example of insect migration is that of the Monarch butterfly *(Danaus plexippus*). The Eastern North American population of Monarchs begins their southern migration in the early fall (September and October) from their summer habitat in the Eastern United States and Canada. Over the next 90 days, individuals will travel up to 4000 km to overwinter in Alpine Oyamel fir forests of the central Mexican state of Michoacán (Fig. 2a; Brower 1996; Urquhart 1987). North American Monarch populations west of the Rocky Mts. make shorter, yet still impressive journeys of up to 1,600 km, travelling from the Western United States and British Columbia, Canada to the California coastline where certain microclimates closely mirror those of the Mexican alpine forests (Fig. 2a; Reppert and de Roode 2018; Yang et al. 2016). With warmer spring temperatures, Monarchs hibernating in the Oyamal forest become active and mate, then begin the migration north. This first generation only reaches the southern United States before females lay their eggs, with the next two to four generations spreading out during spring and summer to re-colonise their summer ranges (Urquhart 1987).

Individual Monarchs only experience a portion of this round-trip journey, meaning each migration is successful despite no previous experience of the route and absent any learned landmark cues. Instead, Monarchs rely on an innate compass to orient, informed by the visual cues of the celestial compass and in particular the position of the sun. Whether Monarchs are ‘true navigators’, with some updating estimate of their current location relative to their goal via a geomagnetic compass, or navigate solely via this celestial-based compass alone is a matter of continued debate (Guerra et al. 2014; Mouritsen et al. 2013a, b; Oberhauser et al. 2013; Reppert and de Roode 2018). Large-scale displacement experiments (Mouritsen et al. 2013a) suggest that Monarchs cannot update their heading direction to the new location, a hallmark of guidance via non-updating compass cues. When migrating Monarchs were experimentally displaced ~ 2500 km from their capture location in Eastern Canada (Guelph, Ontario) and released in Western Canada (Calgary, Alberta), monarchs did not update their headings and continued to fly to the southwest (Mouritsen et al. 2013a).

Monarchs rely primarily on a sun compass (Fig. 3) for both the southward migration in the fall as well as the spring northward recolonisation, with individuals attending to the sun’s azimuthal position (Fig. 3) to maintain their heading (Guerra and Reppert 2013; Mouritsen and Frost 2002; Perez et al. 1997; Reppert et al. 2016). Monarchs’ heavy visual reliance on the sun’s position has been well documented both in headings after release as well as in tethered individuals within flight simulators (Guerra and Reppert 2013; Mouritsen et al. 2013a; Perez et al. 1997; Stalleicken et al. 2005). Use of this compass is widespread in diurnal migrating butterflies with additional evidence coming from the painted lady (*Vanessa cardui*) in its migratory flights between Africa and Europe as well as neotropical butterflies (Guerra and Reppert 2015; Nesbit et al. 2009; Oliveira et al. 1998; Srygley and Dudley 2008). As the sun’s position in the sky varies predictably, moving ~ 15° each hour from East to West throughout the day, the Monarch’s sun compass also contains a compensator mechanism for this movement. The Monarch compensates for this movement and maintains its desired migratory heading via a circadian clock, an oscillator that predictably cycles with the sun’s movement (Froy et al. 2003; Mouritsen and Frost 2002; Reppert et al. 2004; Sauman et al. 2005; Stalleicken et al. 2005). When this natural circadian clock is shifted by exposing individuals to an altered day/night cycle over a number of days, the subsequent migratory heading becomes predictably shifted with this new altered clock. When wild-caught Monarchs were clock-shifted by artificially either advancing or delaying sunrise and sunset by 6 h, headings shifted predictably. Clock-shifted Monarchs showed headings under ambient conditions that were shifted 90°, in line with a circadian clock that had been shifted by 6 h (Mouritsen and Frost 2002). This compensatory mechanism has not been shown to be present across all migrating butterflies, with *V. cardui* migrants only showing evidence of a simple sun compass that does not compensate for time (Guerra and Reppert 2015; Nesbit et al. 2009). It has been hypothesised that time compensation may not be necessary in butterfly migrants that are migrating in the general direction of more favourable climates rather than to specific sites because of the increased leniency for small directional errors. Yet, recent work in dipterans may argue against this conclusion. High altitude day-time hoverfly migrants (*Scaeva pyrastri* and *Scaeva selenitica*) make seasonal migrations to the north (spring) and south (autumn). During spring, these flies rely on favourable southerly winds during their northward migrations to the United Kingdom (Gao et al. 2020). Yet, in autumn, these populations return south against these prevailing winds. Among the mechanisms these migrants employ to counter these flows is the possession of a time-compensated sun compass to maintain correct orientation as they migrate south in the autumn (Gao et al. 2020; Massy et al. 2021). Importantly, these hoverflies travel between broad areas of favourable climate conditions across Europe rather than specific sites, yet still show evidence of time compensation in their compass (Odermatt et al. 2017). These conflicting findings across insect groups underscore the need for further comparative work in non-model species to assess the extent that migrants rely on a simple or time-compensated sun compass.

Insects are known to attend to visual compass information from multiple celestial cues concurrently (Beetz and el Jundi 2018; Wystrach et al. 2014). Beyond tracking celestial bodies such as the sun or moon, insect navigators may also attend to the sky’s polarised light pattern, which can be especially useful when the celestial bodies are visually obstructed by cloud cover or below the horizon (Dacke et al. 2003; Duelli and Wehner 1973; Freas et al. 2017a, 2019a; Lebhardt and Ronacher 2013; Wehner and Müller 2006). Polarised light is detected visually via a specialised region of the insect eye, the dorsal rim area (Homberg and Paech 2002; Labhart and Meyer 1999; El Jundi et al. 2015). In Monarch butterflies, eye physiology as well as intracellular evidence suggest that a polarised light-based compass is likely. The Monarch's eye contains a dorsal rim area that is sensitive to polarised light (Stalleicken et al. 2006). Additional intracellular recordings from the Monarch butterfly's central complex indicate that the sky compass responds to both distinct polarised light patterns as well as the sun’s position (Heinze and Reppert 2011; Nguyen et al. 2021, 2022). Behavioural data is mixed in its support of a polarised light-based compass. When Monarchs were tested with their overhead polarisation pattern rotated 90° off the ambient direction, individuals updated their headings to this new pattern (Reppert et al. 2004). Yet separate testing of this compass produced conflicting results, with Monarchs unable to orient using polarised light cues alone (Stalleicken et al. 2005). Furthermore, Monarchs were able to orient using their sun compass when polarised light cues were absent via covering the eyes’ dorsal rim areas, suggesting these cues were not critical for the time-compensated celestial compass to function (Stalleicken et al. 2005). These results strongly indicate that the sun’s position is the main orientation cue Monarchs use while migrating, while the polarisation pattern may be used as a backup mechanism. As a final note, recent findings (Beetz et al. 2022) of neural activity recordings within the central complex of tethered flying Monarchs suggest that idiothetic cues tied to flight feedback play a dominant role in maintaining an accurate compass representation during movement, allowing Monarch butterflies to maintain their desired compass direction even when visual cues become unreliable.

Another well studied example of sun compass use for maintaining migratory orientation is provided by desert locusts (Fig. 1f). The migratory behaviours of locusts have been recorded for millennia, with locust swarms following seasonal precipitation patterns to make migratory journeys of up to 5000 km. While the migratory patterns of locusts and their underlying navigational mechanisms have received less attention compared to other insect groups, likely due to the presumption that they rely largely on the prevailing wind, a scattered record of behavioural evidence supported by clear physiological and neural evidence is highly suggestive that these animals maintain a sky compass that likely aids their navigation (Homberg 2015).

Locusts are categorised as a group comprising a number (~ 25) of short horned grasshopper species within the family Acrididae which are typically solitary yet aggregate together in migratory swarms when population densities increase (Simpson et al. 1999; Pener and Simpson 2009). Out of this group, one of the most well understood journeys is that of the desert locust (*Schistocerca gregaria*), which travels between northern/eastern Africa and southwestern regions of Asia where it can devastate local agriculture (Devi 2020). These regions are generally arid, with long droughts broken up by periodic precipitation. Environmental changes are tightly correlated with desert locust migratory and reproduction patterns, with rainfall promoting breeding and increases in population density, leading to a phase switch into a gregarious phase where they aggregate into groups and begin migration (Simpson et al. 1999). The migratory journeys of desert locusts rely heavily on moving downstream along wind currents to reach ‘wind convergence zones’ where precipitation is likely (Drake and Farrow 1988). Desert locust migrations tied to environmental changes are theorised to allow populations to rapidly exploit these new habitats that have become more suitable following precipitation events (Dingle 1972; Homberg 2015).

Overall, behavioural evidence collected in the field is suggestive of a reliance on the visual sky compass during migratory movements, though some conflicting evidence and low sample sizes leave much to be desired when making claims based solely on behavioural data (Kennedy 1945, 1951; but also see, Ellis and Ashall 1957). Yet, this scattering of behavioural evidence is strengthened by a number of laboratory-based neural and behavioural findings that corroborate the viability of a sky-based compass for migratory heading maintenance in these locusts. Some of the earliest behavioural evidence of a sun mediated compass in migratory locusts comes from the movements of flightless juveniles. These juvenile desert locusts also conduct directed movements through ground-based swarms similar to the aerial adult swarms called ‘marching hopper bands’. Just as in aerial swarms, these bands move in a fixed direction over multiple days, but their movement is not connected to the wind’s direction, suggesting another compass cue was at play. When the sun’s position around these individuals was experimentally mirrored (shifted by 180°), foragers were observed to shift their orientation in line with the sun's updated position (Kennedy 1945). A similar sun-mirroring experiment was conducted on flying adult locusts with similar observed changes in their orientation, with individuals reorienting in regard to the updated sun’s direction, suggesting that the sky compass also plays a role in orientation during flight (Kennedy 1951). Given the low sample sizes presented in these behavioural studies, this evidence alone would be insufficient to make strong claims regarding celestial cue use. More recent laboratory-based work, however, shows strong behavioural, physiological, and neural support for the use of a sky-based compass in desert locusts (Eggers and Weber 1993; Heinze and Homberg 2007, 2009; Homberg et al. 2011; Mappes and Homberg 2004; Pegel et al. 2018; Schmeling et al. 2014, 2015). Most recently, intracellular recordings of the locust’s central complex inputs suggests that multiple celestial compass information streams (such as polarised light patterns and the sun’s position) are likely tracked in parallel and combined to determine orientation (Takahashi et al. 2022).

### Celestial cue use during nocturnal migration

Many species of moth make long distance migratory journeys at high altitudes similar to migrating butterflies, albeit at night, when the available visual cues change drastically (Chapman et al. 2015; Warrant and Dacke 2011, 2016). Unlike insects that navigate during the day and can rely on the high predictability of the sun’s position to inform their compass, nocturnal navigators face two additional challenges. First, ambient light levels are greatly reduced at night, making detection of visual cues such as terrestrial landmarks more difficult. Second, the available celestial cues at night, such as the moon, are more variable both in terms of their luminance and position, making them less reliable as compass cues for long-term orientation over multiple nights (though see avian models; Emlen 1970). A lunar compass (Fig. 3) appears to, at the very least, not to be critical for orientation in nocturnal moths, with observations in Bogong moths (*Agrotis infusa*), large yellow underwings (*Noctua pronuba*) and silver Y’s (*Autographa gamma*) all maintaining their headings on both moonless nights or when the sky was overcast (Champman et al. 2008b; Dreyer et al. 2018a, b; Warrant et al. 2016). Experimental evidence suggests that another species, the armyworm moth (*Spodoptera exempta*), does not orient via a lunar compass, with tethered individuals in a flight simulator not shown to orient to the moon’s position (Riley et al. 1983). Nocturnal navigation via visual cues is by no means impossible for insects with well documented use of both terrestrial and celestial cues for orientation and homing (Dacke et al. 2003; Dreyer et al. 2018b; Warrant and Dacke 2016). It is theorized in high altitude nocturnal moths that a more stable non-visual cue, the geomagnetic compass, is the primary underlying mechanism for maintaining a migratory heading, with evidence across multiple species (Baker and Mather 1982; Dreyer et al. 2018b; Xu et al. 2017). Despite this reliance on a magnetic based compass, visual cues still clearly play an important role in heading maintenance of high-altitude nocturnal migrants in at least one species, the Australian Bogong moth (*Agrotis infusa*).

Similar to Monarch butterflies, Bogong moths migrate at high altitudes to specific estivation sites, yet Bogongs conduct their migratory journeys at night during the spring in order to escape the high temperatures of the Australian summer (Common 1952). Newly emerged moths journey southward across New South Wales or east across Victoria to reach high altitude estivation caves in the Australian Alps, in journeys that can extend over thousands of kilometers (Fig. 2b, Warrant et al. 2016). In the fall, individuals leave these caves and make the return trip back to their breeding ranges with each generation making the round trip once. Previous experience of the migratory route means individuals could rely on learned cues during their return trip yet migrating to and finding the specific hibernation cave is accomplished by naïve individuals. Bogongs’ long-distance migration requires individuals to maintain a heading direction through the night via either a geomagnetic or celestial compass. Work from Dreyer et al. (2018b) showed Bogong moths using a combination of a geomagnetic compass and visual landmarks to orient during their migratory flights. Tethered Bogong moths flying in a simulator had the surrounding landmarks and the magnetic field rotated either in tandem or in conflict. Individuals remained oriented when the directional distance between the visual and geomagnetic cues remained stable, yet if these cues were rotated to create cue conflict, the moth’s flight orientation broke down. These Bogongs appeared to favour heading directions informed via visual landmarks, yet only when this direction was confirmed by the available geomagnetic cues. Yet, what visual landmarks could such high-altitude migrants attend to during natural migratory flights? Dreyer et al. (2018b) theorised that this interaction between visual and geomagnetic cues could be explained by the geography of the species’ migratory path, with individuals following mountain ranges in New South Wales and Victoria to reach their destination. The tops of these mountains could provide a prominent visual cue at the skyline. While these cues are likely visible even at high altitude, each migrant would pass multiple mountain tops during their journey and thus orientation via these landmarks is only useful while they are confirmed by the geomagnetic compass. As migrants pass by each mountain top landmark, this cue relationship breaks down due to misalignment with the compass and the moth likely chooses a new mountain to set its flight heading to.

The presence of a geomagnetic compass for migratory orientation in the Bogong appears clear, and such a mechanism can readily explain the Bogong’s sustained orientation on overcast nights. However, neither line of evidence rules out the potential for Bogongs to also attend to visual cues such as the celestial compass or celestial cues as short-term reference points, such as the moon’s or prominent star’s position, for heading maintenance when they are available. A recurring theme within insect navigation is that, like many vertebrates, insects often attend to multiple, typically redundant, sensory cues concurrently as built-in backup mechanisms in case one strategy fails (Büehlmann et al. 2020). To date, the only published study concerning celestial cues use suggests that Bogong moth migrants do not orient via the Milky Way (Fig. 3), mimicked by a strip of LED lights positioned above the flying animal (Jansson 2021). Despite the current lack of evidence for celestial cue use in Bogong moths, the potential presence of multiple compass systems based on disparate sensory inputs, with both a visual celestial-based compass and a geomagnetic compass providing heading information, would align the Bogong’s toolkit with the navigational mechanisms that have been proposed for the Monarch, minus the geomagnetic inclination (Guerra et al. 2014; Reppert and de Roode 2018). Such redundant compass systems could explain the successful flight headings on overcast days when celestial cues are obscured in both Bogongs and Monarchs (Dreyer et al. 2018b; Reppert and de Roode 2018).

### Terrestrial cue use during migration

In addition to the visual cues in the sky, it is theorised that Monarchs may also attend to terrestrial landmark cues to direct their migratory flights. In particular, the observed funnelling effect during migration, with migrants avoiding mountains and large expanses of water, may be mediated by the visual features of these prominent landmarks (see Reppert et al. 2010). Monarchs have been observed to exhibit course corrections that occur when individuals reach the Western boundary of their migratory paths, the Rocky or Sierra Madre Oriental Mountains. These heading changes suggest that Monarchs, instead of simply avoiding mountains, may follow the prominent visual cue of these mountain tops by combining them with their celestial compass heading to reach their goal (Calvert 2001). It is currently unknown if migrating Monarchs attend to the cues of the ground and sky concurrently, yet as previously discussed, a similar phenomenon occurs in Bogong moths. Landmark-based orientation in Monarchs might be accomplished by attending to the skyline panorama, a common orientation cue in insects (Franzke et al. 2020; Graham and Cheng 2009; Schultheiss et al. 2016b; Towne et al. 2017). Non-migrating Monarchs can integrate skyline cues and their sun compass, with tethered Monarchs able to weight these separate visual cues when choosing a flight direction (Franzke et al. 2020). Alternatively, when the skyline was presented alone, Monarchs only followed this cue for a short time, suggesting that the skyline aids in flight stabilisation (Franzke et al. 2020). Given that Monarchs combine and weigh multiple visual cue streams during non-migratory movement, such interactions may also occur during migration.

Finally, while the above strategies are capable of transporting migrating Monarchs within a few hundred kilometers of their goal, it remains unknown how they pinpoint their overwintering sites (Mouritsen 2018; Reppert and de Roode 2018). The prevailing theories suggest that olfactory cues likely play a major role, either from the oyamel fir trees or cues left from previous generations of conspecifics (Reppert and de Roode 2018). In the final stages of navigation, these trees may also provide visual beacons, attracting migrants to roost, yet this phase of Monarch navigation remains understudied.

### Wind compensation

Flying migratory insects are often aided in their journey by complementary wind patterns, which can greatly increase the distances these animals can cover. Yet, given insects' small size, wind forces can also present a challenge when their directions do not align with the navigator’s goal. Flying insects are known to adapt to wind forces through multiple responses (Chapman et al. 2011): by selecting periods to migrate when winds speeds are low or by reducing their altitude to near the ground into zones where wind flow is reduced. Additionally, insects are able to actively adapt their headings to compensate for their heading directions, at least partially, in the face of wind displacement (Chapman et al. 2010; Srygley and Dudley 2008). Many butterflies and dragonflies conduct their migratory flights during the day and close to the ground level where wind speeds are slower (flight boundary layer), allowing individuals increased control over both their flight direction and greater ability to compensate for wind drift. Other migrant insect groups, including nocturnal moths, locusts as well as two (uncharacteristic) butterflies, painted lady, and Monarchs, typically migrate at high altitudes where wind speeds are much higher than at ground level and migration direction is often determined by wind direction (Chapman et al. 2015). These migrants rely on strong favourable winds to help propel them in their desired direction, be that their seasonal north/south movements or following rainfall patterns (Brower 1996; Calvert 2001; Gibo and Pallett 1979; Knight et al. 2019; Reppert and Roode 2018). When headwinds are strong, high-altitude migrants regularly respond by descending to the flight boundary layer or ceasing migratory flight to shelter until conditions improve (Calvert 2001; Gibo and Pallett 1979; Stefanescu et al. 2013). Yet even complementary winds are often directionally off from the desired migratory direction by some degree, leading to wind drift that pushes migrants off the desired compass heading, which mechanisms such as the sky compass cannot correct. Given that many insect migrants rely heavily on the solar compass for orientation, how do flying insect migrants compensate for these forces?

High- and low-altitude migrants appear to show distinct wind compensation mechanisms, likely based on the cue availability at their travelling altitudes. First, these animals need some mechanism by which to sense the wind’s flow either through direct sensory input or by assessing the wind’s effect on their migratory path. In high altitude nocturnal moths, non-visual features of wind turbulence are sensed by the antennae in order to assess flow and the current consensus is that detection of visual cues from ground movement is not used at these altitudes (Chapman et al. 2010, 2015; Reynolds et al. 2010; Sane et al. 2010). Similar mechanisms of detecting wind direction are hypothesised for high-flying diurnal migrants such as Monarchs (Reppert and de Roode 2018). However, low-altitude species appear to heavily employ the use of visual cues to sense drift (Srygley et al. 1996; Srygley 2003; Srygley and Dudley 2008). Several low-altitude diurnal dragonfly and butterfly migratory species inhabiting the Panama isthmus migrate between the coastal forests on the North and South coasts, passing over the Gatun Lake. These migrants’ ability to compensate for drift from crosswinds even when flying over open water has been described in Srygley and Dudley (2008). Desert locusts also are able to use ventral optic flow (Fig. 3) to maintain their heading direction (Preiss and Gewecke 1991; Preiss 1992). Crosswind drift is thought to be detected visually in these species primarily through use of the movement of the ground’s features across their compound eye, called ventral optic flow. These insects respond by orienting towards the crosswind to compensate for this lateral displacement, though this compensation may only partially counteract the displacement. Wind detection via optic flow when flying over open water is thought to be more difficult as the water’s surface moves with the wind. This deteriorated visual cue is thought to result in the observed only partial crosswind compensation of dragonflies travelling over Gatun Lake. In contrast, butterflies flying over the lake are able to fully compensate for crosswind drift, suggesting other mechanisms are at play (Srygley and Oliveira 2001). This observed full drift compensation over water is believed to involve the use of visual landmarks along the shoreline, though the exact mechanism is unknown. When such landmarks are absent, such as when the Cloudless Sulphur butterfly (*Phoebis sennae*) crosses the Caribbean Sea, individuals are only able to partially compensate for drift via using the water’s surface as a ground reference, suggesting shoreline landmarks are critical for full crosswind compensation over open water (Srygley 2001).

Migrating desert locust swarms typically move downwind, exploiting the seasonal shifts in the Intertropical Convergence Zone that are highly predictive of rainfall, to carry them to their breeding areas (Chapman et al. 2015; Dingle 2014). The degree to which desert locusts rely on these downwind movements has been the subject of some debate. Some argue that locusts only conduct ‘active downwind orientation’, with individuals matching their heading direction with that of the wind's flow direction (Chapman et al. 2011; Draper 1980). Such a strategy can be useful when attempting to reach a general goal area rapidly yet leave individuals at the mercy of the wind’s direction. Others posit, as discussed earlier in this review, that despite taking advantage of favourable wind directions, locust swarms must possess some level of active compass orientation, evidenced by multiple observations of these swarms deviating from the dominant wind direction, necessitating the presence of a compass (Baker et al. 1984; Chapman et al. 2011; Homberg 2015; Riley and Reynolds 1986). In the context of wind displacement, the active maintenance of a compass heading would aid migratory flights by countering displacements by wind gusts (Chapman et al. 2011; Homberg 2015), though locusts may compensate for wind displacement through other visual strategies such as the optic flow of the ground (Preiss 1992). There is some evidence that the desert locust responds to drift produced by ground pattern movement while tether

ed (Preiss and Gewecke 1991; Preiss 1992). More field-based experimentation along the migratory journey would be useful in untangling these uncertainties (Homberg 2015).

## Homing

Maintaining a heading direction during short goalless orientation bouts only requires the detection of suitable compass cues and to set a desired orientation relative to this compass. The same holds true for migratory flights, at least in the long-distance orientation phase (Mouritsen 2018), with individuals only required to hold a heading direction until they reach a suitable region. During the migratory final phases, some migrants such as the Monarch and Bogong may need to pinpoint specific sites, and given these animals are naïve to these locations, they are theorised to rely heavily on olfactory gradients emanating from the sites to reach their specific goal (Reppert and de Roode 2018; Warrant et al. 2016).

In contrast to these orientation behaviours, during homing, animals return to known locations, commonly their nest/burrow or previously visited resource sites, requiring the animal to learn some aspect of these sites for future use. Homing, in addition to maintaining a desired heading during movement, requires navigators to continuously update an estimate of their current position in relation to their goal, be this their nest or a known resource site. A rich tradition of both lab- and field-based research has long focused on the guidance systems of insects, with a heavy emphasis on hymenopterans, and has revealed that these animals possess a toolkit of concurrently operating navigational systems which underlie orientation and homing behaviours (Collett and Zeil 2018; Collett 2019; Hoinville and Wehner 2018; Legge et al. 2014; Wehner 2020). Within this navigational toolkit, visual cues support three of the major homing mechanisms: path integration and visual landmark memories, and systematic search behaviour.

### Path integration

The path integrator system is a form of dead reckoning in which an estimate, or vector, of both the direction and distance to the start position is continuously updated during the trip, allowing navigators to return to the start directly via their homeward vector rather than retracing an outbound route. Path integration can be useful for foraging or homing insects across a number of spatial scales (Beekman and Ratnieks 2000; Behbahani et al. 2021; Müller and Wehner 1988; Patel et al. 2022). In the case of walking ants, the path integrator typically operates on the scale of meters, with most foraging trips extending between a few to a few hundred meters from the nest. In contrast, flying honeybees (Fig. 1g) maintain path-integration-based vectors for foraging trips that can extend over multiple kilometers (Beekman and Ratnieks 2000). While the directional component is informed via a celestial compass across many insect navigators (Rossel et al. 1978; Rossel and Wehner 1984, 1986; Wehner 1997), the mechanism underlying the navigator’s distance estimate depends on the mode of transport (walking vs. flying). Flying bees rely on optic flow to assess travelled distances (Srinivasan 2011; Srinivasan et al. 1996, 2000) while the primary mechanism for walking ants is a form of mechanoreceptive step-counting (Wittlinger et al. 2006). Path integration is especially useful to navigators when their environment does not contain prominent landmarks or prior to learning the landmark cues of the area. As navigators become more experienced in visually cluttered environments, the path integration system continues to run, yet visual landmark cues start to dominate the navigator’s behaviour. Visual landmarks and path integration constitute the two primary navigational mechanisms for homing in both flying and walking insects as well as across all light conditions (diurnal, crepuscular and nocturnal insects).

#### Path integration in walking insects

Ants have provided an excellent model system for the study of path integration in walking insects and how the path integration system interacts with other homing systems. Ants inhabit a diverse range of visual environments from featureless salt-pan deserts to dense rainforests. These central-place foragers must frequently navigate to find food pieces and return with this resource to their nest. Foragers use multiple sensory cues to conduct these journeys, including visual, geomagnetic, olfactory and idiothetic cues. Ants are widely associated with utilising the chemical cues of a pheromone trail. Yet, while foraging ants can maintain orientation with the trail via tropotaxis (Draft et al. 2018), there is little evidence in ants that straight-line pheromone trails provide directional information (Czaczkes et al. 2015; though see Jackson et al. 2004 for branched trails). This lack of directionality within the pheromone means other cues must resolve this uncertainty for ant navigators to home successfully. Across a number of ant species which search for food alone, either during all or part of their foraging journey, visual cues support the main navigational strategies of these ants (Cheng et al. 2009; Wehner 2020). Additionally, even species which forage on pheromone trails often rely on visual cues to resolve the directional ambiguity of the pheromone (Freas et al. 2019b; Freas and Spetch 2021).

In navigating ants, path integration begins as the forager exits the nest. During each trip away from the nest site, navigators maintain a working-memory-based estimate or vector of the entrance in relation to their current position. This estimate allows ant navigators to return in a straight line to the nest entrance after finding food. While returning to the nest, the path integration system is still operating, resulting in the ant ‘running off’ its vector during the inbound route to the nest, with the system resetting each time the navigator enters the nest (Knaden and Wehner 2006). The distance component of the path integrator in walking ants is informed primarily by a non-visual pedometer or step-counting measurement based on idiothetic cues (Wehner 2020; Wittlinger et al. 2006). However visual cues can still be used to calculate these distance estimates in certain settings. First, *Cataglyphis fortis* foragers have been shown to respond, albeit weakly, to ventral optic flow in addition to their step counting mechanism to estimate distances (Ronacher and Wehner 1995) while ignoring lateral based flow (Ronacher et al. 2000). Given these findings, the stride integrator appears to dominate the distance estimate of path integration, with only minor influence from optic flow. Yet, optic flow is sufficient alone to form distance estimates in some cases. During social transport in *Cataglyphis bicolor,* when individuals were carried by nest-mates during an outbound segment and then separated, carried individuals followed a vector accumulated during this transport despite the lack of a pedometer (Pfeffer and Wittlinger 2016). Similar to other insects, ants possess a celestial compass which informs the directional component of their path integrator. Unlike the celestial compass in many flying lepidoptera, where the sun’s position provides the primary celestial cue for orientation, in ants the path integration system is heavily informed by the sky’s polarisation pattern, especially when the sun’s position is occluded (Freas et al. 2017a, 2019a; Lebhardt and Ronacher 2013; Reid et al. 2011; Wehner and Müller 2006).

While path integration while walking on the ground has primarily been studied in ants, other Hymenoptera members, including honeybees (*Apis mellifera*) and bumblebees (*Bombus terrestris)* show evidence of path integration accumulation while walking. Early work in walking honeybees, focusing on the characteristics of their waggle dance (see navigational communication section below) found that walking honeybees accumulated a path integrator very similar (though scaled down) to their flying counterparts (Bisetzky 1957). More recently, path integration in walking bumblebees (Fig. 1h) has been characterized in Patel et al. (2022). When bumblebee foragers in the lab foraged from a feeder only accessible by walking, these foragers encoded both distance and directional estimates of the return trip. When overhead cues, mimicking celestial cues such as the polarisation pattern and sun’s position were rotated, foragers’ inbound paths were correspondingly shifted. How distances were encoded in this paradigm remains unclear, though optic flow and stride integration (like in walking ants) are the likely mechanisms (Patel et al. 2022).

#### Path integration in flying bees and wasps

Path integration has also been studied in flying insects such as honeybees, wasps and fruit flies. Bees and wasps often extensively search their habitat for new food sites on the outbound portion of their foraging trip, resulting in long, winding routes. Yet, via path integration these flying navigators can return to their home location directly, similar to feats observed in ants. As described above, the path integrator allows walking or flying navigators an updating estimate of their origin location while foraging that couples a directional compass and a distance estimate. The directional component of the path integrator appears to be largely identical in both honeybees and ants with celestial cues playing the primary role. The retina of honeybees contains a dorsal area that is highly sensitive to polarised light (Labhart 1980; Labhart and Meyer 2002; Wehner and Strasser 1985). Additionally, behavioural evidence in both forager flight paths as well as their waggle dances in the hive indicate that a celestial compass is the primary directional cue for the path integrator (Dacke and Srinivasan 2008; Evangelista et al. 2014; Rossel and Wehner 1984, 1986).

The clear mechanistic difference of path integration between walking and flying insects is how these navigators estimate distances. While walking ants rely primarily on a stride integrator, which is sensed non-visually, flying insects cannot rely on wingbeats for accurate estimates given the variabilities in air flow. Instead, flying insects rely solely on visually based optic flow to calculate distance travelled. This optic flow mechanism during homing, demonstrated largely in honeybees, operates somewhat similarly to that of migrant insects compensating for wind drift (discussed above), with navigators assessing the speed of visual cues that move across their retina as they move through an environment (Preiss and Gewecke 1991; Preiss 1992; Srinivasan et al. 2000; Srygley and Dudley 2008). Unlike migratory journeys where this visual movement is used to compensate for wind displacement without informing the navigator of its current position, in homing insects optic flow is integrated into updating their path integrator system. This allows foragers to complete the return portion of their foraging trip as well as store long-term memories of locations of profitable resource sites for future trips.

### View memories

The environment surrounding a home nest often provides a variety of landmarks that may range across several sensory modalities (olfactory, visual, magnetic, and vibrational). Extensive research has shown that ant navigators are adept at learning these landmarks and hence they have been an excellent model species to study how sensory cues guide homing behaviour (Büehlmann et al. 2020; Wehner et al. 2006; Wehner 2020). Across a number of well-studied ant species, learning the visual cues of the full terrestrial panorama (Fig. 3) around goal locations and along the foraging route is relied upon heavily for successful navigation (Freas and Cheng 2018, 2019; Freas et al. 2021; Graham and Cheng 2009; Mangan and Webb 2012; Narendra et al. 2013; Wehner 2020; Zeil and Fleischmann 2019). Ant navigators are thought to rely on this panoramic scene rather than any individual landmarks as their vision is characterised by a wide visual field (~ 300°) coupled with low visual acuity, making discerning the characteristics of individual landmarks difficult (Schwarz et al. 2011).

The use of view memories has been modelled through several mechanisms, yet the aspects of the panorama employed remain debated. Most models involve view matching, where view memories are acquired during multiple pre-foraging learning walks as well as during the establishment of the foraging route. Retained view memories are then compared to the navigator’s current view during subsequent foraging trips to direct movement to goal sites. A number of mechanisms for this comparison have been proposed, including pixel matching (Zeil et al. 2003), view familiarity (Baddeley et al. 2012), view prediction (Möller 2012), assessing the fractional position of landmark masses (Lent et al. 2013), and the UV contrast between the ground and sky composing the skyline (Freas et al. 2017b; Graham and Cheng 2009; Schultheiss et al. 2016b) and the ‘copy-and-shift’ neural model (Sun et al. 2020). Importantly, many of these view-based navigation models have typically only focused on view comparisons in which views are deemed attractive, resulting in forward movement. Yet recent lines of evidence in multiple desert ant species (*Melophorus bagoti*, *Cataglyphis fortis* and *Cataglyphis velox*) have characterised evidence of view memories that are aversive, causing foragers to cease forward movement and turn away from the associated directions (Freas et al. 2022; Schwarz et al. 2020; Wystrach et al. 2020). This evidence has led to the expansion of view-based navigation models to include aversive views (Le Möel and Wystrach 2020; Murray et al. 2020). The interplay of learned attractive and aversive views has been hypothesised to allow quick decisions regarding navigation, deciding to turn away or move forward when presented a single view rather than sampling multiple views.

Newly emerged ants do not immediately leave the nest to begin foraging, as individuals first need to learn the panorama surrounding the nest entrance and to calibrate their path integrator (Zeil and Fleischmann 2019). During the first few trips outside the nest, individuals conduct multiple learning walks in different directions around the nest. These short trips typically number between three to seven and are characterised by foragers travelling in small loops extending away from the nest a short distance before returning to the entrance. When species inhabit visually rich environments, observed in *Cataglyphis* and *Myrmecia* species, these learning walks contain two elements, pirouettes, and voltes. Pirouettes are rotational scans where the individual stops and rotates in place, stopping intermittently during rotation. The longest stop of the ant’s rotation during a pirouette occurs when they are aligned with, or facing, the nest entrance, suggesting this is a period when the navigator is acquiring views of the nest panorama (Fleischmann et al. 2017, 2018b; Zeil and Fleischmann 2019). Voltes, in contrast, occur when the individual makes a small loop along their journey with no stops. In species where the nest panorama is visually barren, such as *Cataglyphis fortis,* learning walks do not contain pirouette behaviours (Fleischmann et al. 2017; Zeil and Fleischmann 2019). Despite the lack of pirouettes observed when environments lack visual landmarks, *C. fortis* still learns visual landmarks around the nest when they are present (Wehner 2008).

The importance of pre-foraging learning walks to visual cue acquisition and orientation near the nest is well established (Zeil and Fleischmann 2019). Yet are the walks themselves supported by visual compass cues? Orienting to the nest during learning walks, before these naïve ants have learned the visual scene around the nest, requires some compass cue. Traditionally, it was believed that the celestial compass portion of the path integrator directs the individual to the nest direction, even during the ant’s first trips. However, when the overhead celestial cues were blocked, European *Cataglyphis noda* ants were still observed to correctly orient to the nest direction (Grob et al. 2017). Instead, a magnetic compass underlies nest-ward orientation and facilitates the acquisition of the nest panorama in naive foragers (Fleischmann et al. 2018a). As these ants become more experienced, they begin to switch to celestial-compass-based orientation. Several intriguing questions remain unresolved regarding visual cues during these learning walks. How much experience is required for foragers to switch from magnetic to visual based orientation? Additionally, whether this magnetic compass underlies nest orientation during learning walks in other ant species has yet to be determined.

View memories acquired during learning walks enable foragers to orient to the nest direction while foragers are within a certain distance range around the nest (Fleischmann et al. 2018b). This range, or catchment area, at which orientation via nest-area-acquired view memories is dependent upon each nest’s local environment (Baddeley et al. 2012; Murray and Zeil 2017; Zeil et al. 2014a). In highly cluttered environments, such as the dense forest in which the Australian bull ant *Myrmecia midas* nests (Fig. 1e), local cues can quickly obscure prominent landmarks as foragers move away from the nest, leading to a decrease in panorama similarity and subsequently decreased forager orientation success over short distances (Freas and Cheng 2019). Small view memory catchment areas are also observed in open desert environments. The individually foraging Sonoran Desert ant *Novomessor cockerelli* typically inhabits cluttered environments, yet these deserts contain few prominent terrestrial cues with even distant mountain ranges being inconspicuous to the ant’s view (Freas et al. 2021). The lack of prominent terrestrial cues that remain stable in the Sonoran Desert results in small catchment areas and subsequently *N. cockerelli* foragers are unable to successfully orient when displaced only a handful of meters away from known locations (Freas et al. 2021). When environments do contain prominent landmarks that remain unobstructed by local clutter and are visible over larger distances, as has been characterised in *M. bagoti*, *C. noda* and *M. croslandi*, the panorama maintains a high degree of similarity as the ant moves away from a known location, facilitating correct orientation over longer distances (Fleischmann et al. 2018b; Murray and Zeil 2017; Narendra et al. 2013; Wystrach et al. 2012). Across all these species inhabiting a variety of visual habitats, the catchment area of nest-area-acquired views during learning walks only supports orientation within 10 m of the nest, yet ant foragers travel much longer distances in the search for food, necessitating the acquisition of additional nest-aligned views along the foraging route.

Beyond learning the nest panorama during learning walks, ant navigators learn multiple panoramas while foraging, both around profitable resource sites and along their foraging routes (Freas and Spetch 2019; Freas et al. 2019a; Islam et al. 2020; Schultheiss et al. 2016b; Wystrach et al. 2019, 2020). In contrast to learning walks, considerably less is known regarding the mechanisms that occur during the first foraging trips that facilitate view learning, especially those of non-nest aligned views. During the first few trips away from the nest area, foragers will occasionally stop and look back towards the nest, with this behaviour disappearing as the forager becomes more experienced, likely making these periods when foragers acquire nest-aligned views along the route (Mangan and Webb 2012; Nicholson et al. 1999; Zeil et al. 2014a, b). In contrast to learning walks, these turn-back behaviours are probably supported by the celestial compass portion of the path integrator, yet more study of learning during these periods is needed. Differences in view acquisition rates in two desert ants, *M. bagoti* and *C. velox*, with foragers of both species showcasing stronger view learning on the outbound portion of their route, provides support to the theory that these turn backs support view learning (Freas and Cheng 2018; Freas and Spetch 2019). Here, foragers learned nest-aligned views after only a single exposure while on the outbound segment of their foraging trip, even when these views shared no similarity to the nest panorama. In contrast, when these foragers only experience these views while travelling back to the nest, acquisition takes multiple trips (Freas and Cheng 2018; Freas and Spetch 2019). Once acquired, view memories remain remarkably stable, allowing for successful orientation over periods of multiple days without repeated exposure (Freas and Spetch 2019; Narendra et al. 2007; Ziegler and Wehner 1997).

View-memory learning around profitable food sites and along the foraging route relies heavily on reinforcement learning. Attractive views, aligned with goal locations, are thought to be reinforced by two outcomes along the foraging trip. First, through the discovery of food during the outbound trip and once again after finding and re-entering the nest at the end of the inbound route. While these outcomes both represent positive reinforcing events, the differences in learning on the inbound and outbound segments indicate that a forager discovering food represents a stronger positive reinforcement than reaching the nest, resulting in stronger view learning during the outbound segment (Freas and Cheng 2018; Freas and Spetch 2019). As mentioned above, evidence in *Cataglyphis* and *Melophorus* suggests that view memories are stored along with a positive or negative valence through associative learning, with positively associated views resulting in forward movement and negatively associated views resulting in hesitations and turning away from these views (Freas et al. 2022; Wystrach et al. 2020). The valence of view memories can change based upon the ant’s current motivational context (Schwarz et al. 2020), or when the views are associated with negative outcomes (Wystrach et al. 2020). The overall valence associated with memorised views is constructed through the accumulation of experiences at the location, with each new experience regulating the memories’ valence based on a prediction-error rule (Freas et al. 2022). Retaining the valence of views along the foraging route is theorised to provide multiple functions for the ant navigator. First, it allows for rapid navigational decisions (move forward or turn) based on a single view comparison rather than sampling multiple view directions (Le Möel and Wystrach 2020; Murray et al. 2020). Additionally, the interplay between attractive and aversive views supports changes in foraging routes detouring around areas associated with negative outcomes (Wystrach et al. 2020). If changes along an established foraging route occur, resulting in the route becoming difficult or impassable, these negative experiences will increase turning behaviours, increasing the chances that foragers avoid this outcome. These new views around the negative outcome are then positively reinforced and result in these newly attractive views forming a new route.

The remarkable homing abilities of bees and wasps have also inspired a long line of scientific inquiry into the role of view memory, with studies stretching back well over a century (Collett et al. 2013; Collett and Zeil 2018; Fabre 1882; Heinze et al. 2018; Stürzl et al. 2016; Zeil et al. 2014b). Similar to walking ant foragers, these flying insects make repeated journeys away from their nests to search for resources and upon discovery, then return with this back to the nest. The ability to home both back to the nest location, as well as to known resource patches relies heavily on visual based cues in these flying navigators. Some of the earliest work regarding how these insects home successfully involved long-distance displacement (Fabre 1882), with a number of bee and wasp species observed to be able to successfully return home after being displaced multiple kilometers from their nests. Successful homing occurred even when the displacements involved transport in darkness, extensive detours along the displacement route, or the removal of the navigator's antennae (Collet et al. 2013; Fabre 1882). Similar early displacement experiments were conducted by Romanes (1885) in honeybees, which heavily suggested these navigators acquired some level of knowledge regarding the landmarks in an area around their hives. Romanes displaced marked honeybees in several conditions, including within the bees' known foraging areas as well as out into the ocean beyond visual landmark range. Marked bees were observed to successfully return to the hive only when displaced within moderate distance. Homing was largely unsuccessful when honeybees were displaced well beyond their home range, either 300 m away or when displaced to the open ocean. These early observations prompted researchers to theorise that these insects must learn some information regarding the visual landmarks around their homes and use it to home back after displacement (Romanes 1885). This theory of landmark use was later substantiated by field research in both wasps and bees using the transformational approach (Cheng and Spetch 1998). Here, prominent landmarks around the wasp or bee’s nest/hive were displaced and the navigator's search location was observed (Tinbergen 1932; von Frisch 1953). Tinbergen studied how solitary wasps (*Philanthus triangulum*) returned to their nest via visual landmarks, finding that by displacing local landmarks (pinecones) around the nest while the wasp was away caused it to search for the nest, the wasp searched for the nest at the landmark's updated location. von Frisch found similar evidence of visual landmark use in honeybees, displacing coloured panels from around the hive with returning foragers following these displaced landmarks to find the hive.

### Systematic search

View memories and path integration are robust navigational strategies that guide returning foragers to the general area close to their current goal. Yet, this does not guarantee the navigator can immediately find the goal as some locations, such as a nest entrance, can be inconspicuous. Additionally, navigational systems are prone to error, meaning that the ant’s view memory and path integrator may not point to the exact goal location, necessitating an ensuing period of search to pinpoint the goal (Wehner and Srinivasan 1981). Studies in desert ants (*Cataglyphis fortis*, *Melophorus bagoti*, *M. oblongiceps*) characterise this search behaviour as systematic in its structure, consisting of loops of increasing size and informed by the continuously running path integrator, the surrounding panorama as well as the search’s onset location (Müller and Wehner 1994; Schultheiss et al. 2015; Wehner and Srinivasan 1981). This onset of search location is useful as the navigator first arrived at this location by following its other navigational strategies, making it the likely location to either find the goal or further directional information upon future passes through the site (Schultheiss et al. 2015, 2020). These occasional returns to the start location result in a looping search path with the magnitude of these loops increasing over time, expanding the search area. These expanding looping paths appear to maximise the probability of finding the nest (Heinze et al. 2018; Müller and Wehner 1994; Schultheiss and Cheng 2011; Schultheiss et al. 2015; Wehner and Srinivasan 1981).

Visual cues can mold the characteristics of these searches during search as well as informing the navigator’s degree of navigational uncertainty that exists prior to its onset. The search spirals of *Cataglyphis fortis* have been shown to be influenced by their preceding vector length (Merkle and Wehner 2010). As the length of the preceding vector was increased, so did the spread of the subsequent systematic search. This increase in spread is indicative of an increase in navigational uncertainty due to the higher accumulation of error in the path integration system over longer vector distances. Similar increases in search spread due to error accumulation in the path integrator have been observed in *Melophorus bagoti (*Fig. 1d*)*, despite an abundance of visual landmarks along the foraging route (Schultheiss and Cheng 2011). These results indicate that foragers maintain an estimate of uncertainty of its current location, that not only dictates when the forager abandons homing and starts to search but also influences the structure of the search itself. The presence of visual terrestrial landmarks in particular has been shown to reduce uncertainty during search. In the Australian desert ant *Melophorus bagoti*, systematic search paths are highly focused with little spread when the surrounding visual scene is rich, while in a visually barren environment, systematic search spread increases (Schultheiss et al. 2013). Importantly, some of this highly focused searching when visual landmarks were present could be the result of foragers switching to view-based navigation rather than systematic search exclusively. Further work in both *M. bagoti* and the Australian salt pan ant *Melophorus oblongiceps* has shown that the presence of landmarks along the homeward route can influence search onset as can the amount of spread in an ant’s search (Schultheiss et al. 2016a). When familiar visual landmarks along the foraging route were removed, *M. bagoti* began their search after running off a shorter distance of their vector while *M. oblongiceps* travelled similar vector distances in the presence or absence of familiar landmarks during training. These differences suggest a heavy reliance on the path integrator in calculating navigational uncertainty in *M. oblongiceps* compared to *M. bagoti*. This dominance, however, can be highly dependent on each nest’s local environment (Büehlmann et al. 2011; Cheng et al. 2014). Despite this difference, both species exhibited an increase in search spread when visual landmarks were removed, suggesting that visual cues showed some influence on the amount of navigational certainty during search in both species (Schultheiss et al. 2016a).

## Navigational communication

**Fig. 4:**
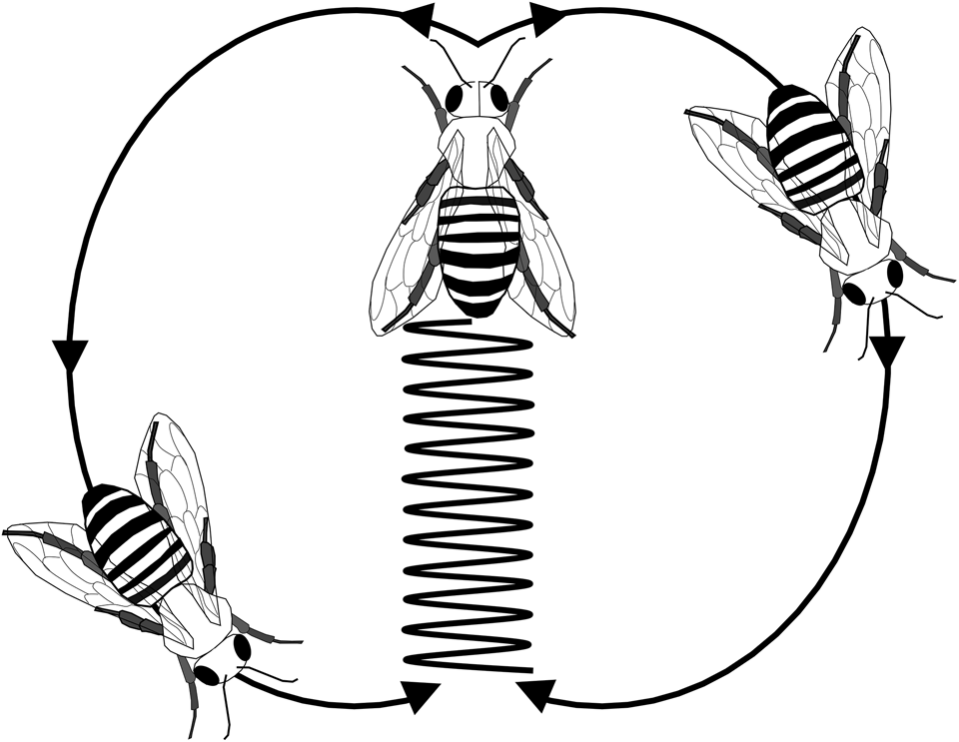
Diagram of the waggle dance. The dancer performs a repeated figure eight while recruited follower bees observe the dancer. Dancers will conduct the central waggle phase repeatedly while the outer loops restart the dance. Duration of the waggle phase communicates the distance of the food source while the food’s direction is tied to the angle of the waggle phase, representing the food’s direction relative to the sun’s azimuthal position

A particularly interesting aspect of homing in honeybees is the communication of visually derived vector information to hive-mates. Path integration information of the outbound path to a profitable patch of food can not only be recalled for the individual’s future foraging trips but this visual information can also be communicated to nest-mates through the honeybee’s waggle dance (von Frisch 1965) as well as information regarding the site’s quality (Seeley et al. 2000). Aspects of these dances, namely the angle and duration of the waggle phase, inform nest-mates of both the direction and distance through communicating the visual cues of the outbound vector. If a honeybee discovers a particularly profitable resource patch while foraging, it can communicate its path-integration information from this trip to its hive-mates through these waggle dances (Barron and Plath 2017). Dances are typically performed while oriented vertically and their shape somewhat resembles a figure eight, with two looping ends and a central zig zagging ‘waggle phase’ (Fig. 4). Dancers will conduct the waggle phase repeatedly with the subsequent loops bringing them back to the beginning where the waggle phase starts again. The duration of the waggle phase communicates information regarding the distance from the hive of the resource site (calculated via optic flow; Collett 2019), while the direction to food is communicated via the angle between the vertical direction and the dance’s waggle phase, representing the food’s direction relative to the sun’s azimuthal position (von Frisch 1967). These dances are illustrative of how visual cues are communicated to others in the navigational context, and help researchers understand how these honeybees calculate their path integrator and the relationship between individual visual memories and communally shared vector information (Dacke and Srinivasan 2008).

Untangling the language of the waggle dance shows that the honeybee’s personal path integration system calculates direction and distance similarly to that of walking ants, with the presence of the celestial compass being critical. In both walking ants and flying bees, the distance estimate only accumulates while their celestial compass is unobstructed. When access to these overhead celestial cues is blocked, both honeybees and ants do not accumulate personal vector information (Dacke and Srinivasan 2008; Sommer and Wehner 2005). This lack of an accumulating distance estimate suggests that distance and direction estimates are stored as an integrated memory rather than as separate memories. Interestingly, honeybees appear to accumulate multiple path integration memories, with a personal distance estimate that accumulates only when the celestial compass is visible and a communal distance estimate which runs continuously regardless of the celestial compass and is communicated to hive-mates through the waggle dance (Dacke and Srinivasan 2008).

Additionally, while visually derived path integration information is understood to be communicated via the waggle dance to naive hive-mates, it also contains multiple other components beyond broadcasting this location (Grüter and Farina 2009). Of these, the most relevant for this review is the activation of personal navigational memories, comprising a number of visual and non-visual strategies, in experienced hive-mates while at the hive (Biesmeijer and Seeley 2005; Reinhard et al. 2004). Once a honeybee forager has experience of a resource site it can rely on its own individually accumulated knowledge, through its vector memory, view-based matching, colour memories or olfactory cues, to make repeated return trips to these sites. When foraging to these sites is interrupted by the flower’s bloom cycle or overnight when foraging ceases, these navigational memories can be recalled when exposed to these site-specific scents on a dancing hive mate. These experienced honeybees will often only follow the dancer briefly and after they exit the hive will rely on their own personal navigational memories to travel to the site and not use socially transferred site information (Biesmeijer and Seeley 2005). Given that during the spring and summer months many of the hive’s foragers are highly experienced, this memory recall activated by the dance may encompass a large majority of hive mate interactions (Biesmeijer and Seeley 2005; Grüter et al. 2008).

## Conclusions

In the past few decades, considerable strides have been made in our understanding of animal cognition, including how animals orient and navigate through their environments. Here we have reviewed how visual guidance systems are employed by insect navigators, focusing on three types of directed movement; short distance orientation, long-distance migratory orientation, and homing behaviours. Across these forms, the use of visual cues plays a large role in many groups, with the use of a celestial compass being the most widespread. Additionally, insects rely on terrestrial landmarks in multiple navigational contexts for either directional or distance information.

As illustrated by examples from a few well-studied model species from different insect orders, navigational mechanism in insects share many commonalities with birds and mammals. For example, many insect navigators use celestial cues to orient, both over long and short distances, with or without a specified goal (Dacke et al. 2021; Warren et al. 2018; Mouritsen 2018). Use of these cues is often combined, sometimes in a Bayesian fashion (Khaldy et al. 2022), with other visual or non-visual cues (Beetz and el Jundi 2018; Büehlmann et al. 2020; Freas and Cheng 2022). Additionally, small insect navigators are often subjected to forces such as wind gusts that could blow them off course. Yet, these insects possess a number of strategies to counteract these forces, including the use of visual cues (Preiss 1992; Srygley 2001, 2003). Many insect navigators also attend to a path integrator when homing that is based on celestial cues and a distance estimate informed primarily by optic flow during flying (Srinivasan et al. 1996, 2000). Perhaps the most interesting feature of insect navigation is the adaptability and flexibility with which cues are used. Information from different sources is often combined and weighted according to reliability (Büehlmann et al. 2020; Wehner 2020). Guidance and attraction to different cues is adapted to the ecology of the species and the typical environment in which they navigate as shown by comparative studies of dung beetles and ants (Dacke et al. 2021; Wehner 2020). Even in cases where certain cues are dominant, there are typically back-up systems that allow the insect to navigate when the dominant cue is unavailable such as in the sky polarisation pattern during overcast conditions or when homing insects conduct systematic search to pinpoint goal locations (Chapman et al. 2008b; Dreyer et al. 2018a, b; Schultheiss and Cheng 2011; Schultheiss et al. 2015; Warrant et al. 2016). Moreover, individual learning experiences can sometimes alter the valence of different cues as in the aphid-associated cues for ladybeetles or the view memories of homing ants (Freas et al. 2019a; Wang et al. 2015; Zeil and Fleischmann 2019).

Thus, like vertebrates, insects show a range of sophisticated and fascinating strategies for solving the spatial navigation problems faced in their lives. Moreover, many navigational strategies are analogous to those used by vertebrate species. Such widespread use suggests that successful means of orienting and navigating have persisted across evolution, yet these mechanisms have also been refined to the local environment with both population and individual experiences shaping the use or weighting of these cues. In addition to demonstrating the breadth of navigational processes across animals, the study of navigation in insects offers several benefits. First, the lifespan of some insects, such as *Drosophilia*, is relatively short, facilitating the study of genetic mechanisms. Second, for some insects such as ants, the small size means that long distance travel (relative to body size) can be easily tracked, and importantly that access to visual and other cues can be modified in the natural environment. Insects’ relatively simple nervous system compared to many vertebrate species has led to a wealth of research on the neural mechanisms of navigational behaviours (see reviews by Le Moël and Wystrach 2020; Heinze et al. 2018; Webb and Wystrach 2016) and has inspired the development of machine learning models of navigation. Finally, research on insect navigation has made important contributions to the goal of illuminating “the evolution of intelligent behaviour and intelligent systems from invertebrates to humans” (Czeschlik 1998).

## Data Availability

No new original data are reported within this review article. There are no data or materials from experiments reviewed here reposited in association with this article.
